# Impact of Stroke History on Cognitive Function, White Matter Hyperintensities, and Circulating BDNF Levels

**DOI:** 10.34172/aim.34390

**Published:** 2025-11-01

**Authors:** Farnaz Hashemi, Saeed Malihi-Alzakerini, Shima Shakiba, Hossein Poustchi, Reza Ghanbary, Maryam Sharafkhah, Shahram Oveisgharan

**Affiliations:** ^1^Department of Health Psychology, Karaj Branch, Islamic Azad University, Karaj, Iran; ^2^Department of Clinical Psychology, University of Social Welfare and Rehabilitation Sciences, Tehran, Iran; ^3^Liver and Pancreatobiliary Diseases Research Center, Digestive Diseases Research Institute, Tehran University of Medical Sciences, Tehran, Iran; ^4^Gene Therapy Research Center, Digestive Diseases Research Institute, Tehran University of Medical Science, Tehran, Iran; ^5^Rush Alzheimer’s Disease Center, Chicago, IL, USA

**Keywords:** Circulating BDNF, Cognitive function, Stroke, White matter hyperintensities

## Abstract

**Background:**

The present study aims to investigate the impact of stroke history on cognitive function, white matter hyperintensities (WMHs), and circulating brain-derived neurotrophic factor (BDNF) levels in brain lesion patients.

**Methods:**

In this study, we enrolled 228 individuals exhibiting clinical symptoms of stroke from the Golestan Cohort Study. The participants were categorized into two groups based on their stroke history. Subsequently, 120 patients with a history of stroke and 108 patients without obvious brain lesions were subjected to comparative analysis using magnetic resonance imaging (MRI). Montreal Cognitive Assessment (MoCA) and Fazekas scores were used to evaluate cognitive function and WMH burden, respectively. In addition, circulating BDNF levels were measured using the Human BDNF Elisa kit.

**Results:**

Totally, 228 patients were recruited in the study with a mean age of 63.8 years. Stroke was found in 52.6%. MoCA scores and plasma BDNF levels were significantly lower in patients with a history of stroke compared to people without such a history after adjusting for age, sex, education and type of residency (adjusted regression coefficient (RC) (95% CI)=-4.0 (-5.0 to -3.0), -3.2 (-4.2 to -2.2), respectively). In addition, the intensity burden of white matter was higher in the stroke group (adjusted RC (95% CI)=1.2 (0.8 to 1.6).

**Conclusion:**

The study suggests that a multi-biomarker approach, encompassing measures such as the MoCA score, Fazekas score, and circulating BDNF levels, can provide valuable insight into the neurological status of post-stroke patients and highlight potential avenues for improving patient outcomes through early detection and intervention strategies.

## Introduction

 Stroke is a destructive cerebrovascular disease that affects more than 15 million people worldwide every year.^1^ Between 1990 and 2019, there was a notable increase of approximately 70% in the incidence of stroke, as well as an over 40% rise in related mortality rates globally.^2^ It has been shown that stroke is the second leading cause of death and the third leading cause of disability globally.^3,4^ Etiologically, stroke can be classified into two categories, ischemic and hemorrhagic, while their clinical manifestations in patients are mostly the same regardless of the cause. Ischemic stroke, which includes approximately 70% of all strokes, is caused by vascular occlusion to the brain, while hemorrhagic stroke occurs due to the rupture of blood vessels within the brain.^3^ Despite all the recent advances in the diagnosis, treatment and management of strokes, its complications are still increasing and have become a major concern for public health worldwide.^5^

 Brain-derived neurotrophic factor (BDNF), a member of the family of neurotrophin growth factors, plays an important role in the development, differentiation and survival of neurons in the central and peripheral nervous system.^6^ Through regulating synaptic function, modulating the release of neurotransmitters, and supporting the growth, differentiation and survival of neurons, BDNF can increase neural plasticity.^7^ It has also been demonstrated that this factor plays a role in regulating mood, anxiety and normal cognitive function,^8^ as well as the prognosis, pathogenesis and rehabilitation of stroke.^9^ Many studies have indicated the role of BDNF after stroke as a prognostic biomarker. Emerging evidence suggests that the level of BDNF in patients with a history of stroke is significantly lower than healthy individuals, and also the severity and type of stroke can probably be related to the level of BDNF.^10-13^

 The Montreal Cognitive Assessment (MoCA) is a widely utilized screening tool to detect cognitive impairment and dementia in their early stages. The MoCA evaluates various cognitive domains, including attention and concentration, visuospatial skills, calculation, executive functions, abstraction, language, memory, and orientation.^14,15^ On average, the completion time for this test per patient ranges from 10 to 20 minutes; additionally, it has been translated into multiple languages for broader accessibility.^16,17^ Moreover, MoCA has been also used in research to identify alterations in cognition over time, and to evaluate the effectiveness of cognitive interventions.^18^ Stroke is one of the leading causes of cognitive impairment, as up to 70% of individuals who have suffered from strokes may present some form of cognitive dysfunction.^19^ MoCA is used as a valuable tool to assess cognitive function in stroke patients, and changes in MoCA scores after stroke may reflect stroke severity and post stroke interval. MoCA scores may also be influenced by factors such as depression and lesion location.^20,21^

 The Fazekas scoring system is a widespread approach used to evaluate white matter hyperintensities (WMHs) on magnetic resonance imaging (MRI) of the brain. WMHs are commonly observed in the elderly population and have been associated with several neurological disorders such as dementia and stroke. The reliability of Fazekas scores has been demonstrated to be high among raters, and they are employed in both clinical and research settings to evaluate the extent and severity of WMH.^22,23^ The Fazekas score is a commonly used visual rating scale to classify the severity of WMH, and determining Fazekas scores after stroke may provide insight into the underlying mechanisms associated with brain damage following stroke.^24^

 In the present study, to improve prognostic and therapeutic strategies for stroke patients, as well as to gain a comprehensive understanding of stroke-related complications and outcomes, we carried out a comparative analysis of “Fazekas scores”, “MoCA scores”, and “plasma BDNF levels” between individuals having a history of stroke (within the range of 5 years to 6 months) and those without. Our research aims to provide insight into the pathophysiology underlying stroke-related complications and outcomes while identifying potential biomarkers that could facilitate early detection and intervention efforts.

## Materials and Methods

###  Study Design

 The Golestan Cohort Study was initiated in January 2004 in northeastern Iran, with a focus on understanding the factors contributing to esophageal squamous cell carcinoma in the high-risk region of the Golestan province. Over the period from January 2004 to June 2008, a total of 50,045 individuals aged 40-75 years were enrolled in this longitudinal study. Participants were followed up annually to monitor health outcomes.^25^ In the follow-up conducted in 2021, 300 individuals with clinical suspicion of stroke within the past 5 years to 6 months before the date of visit were invited to participate in the current investigation. The purpose of this study was explained to patients who were suspected of having a stroke by trained personnel via phone calls. Ultimately, 228 individuals consented to participating in the present study. All participants underwent brain MRI scans as well as cognitive evaluations. Additionally, blood samples were collected from all patients and stored for future analysis.

###  Study Population

 For the current study, 228 patients were enrolled based on presenting clinical symptoms suggestive of stroke and suspected stroke diagnosis. All patients underwent blood tests, MRI scans, cognitive function assessments, and psychological evaluations. Subsequently, all patients were referred to a designated center for MRI scans. After the MRI results were analyzed by a specialist radiologist and neurologist, it was noted that only 120 patients exhibited evident brain lesions and signs of stroke and the size and location of the stroke were determined for them, while the remaining 108 participants were designated as the control group for comparative analysis purposes. None of the individuals in the control group displayed notable brain lesions on MRI examination. All patients provided written informed consent before undergoing cognitive testing. Inclusion criteria for the study involved providing informed consent and acquisition of blood draw. Exclusion criteria comprised refusal to proceed with further evaluations and exhibiting signs of mental retardation in the Golestan Cohort records.

###  Cognitive Function (MoCA) Test

 This assessment comprises a 30-point test that fits on one side of an A4 sheet and can be completed in approximately 10 minutes. The MoCA evaluates various cognitive areas, including Visuospatial/Executive function, Naming, Memory, Attention, Language, Abstraction, Delayed Recall, and Orientation (temporal and spatial).^26^ The Persian translated version of MoCA was administered to all patients. The reliability of this test was previously reported as 92% using Cronbach’s alpha coefficient method.15 The MoCA is scored on a scale of 0 to 30 points. Scores are classified into the following categories: normal cognition (26–30), mild cognitive impairment (18–25), moderate cognitive impairment (10–17), and severe cognitive impairment (scores below 10).^26^

###  Grading of White Matter Lesions (Fazekas Scoring)

 The severity of WMHs was evaluated using the Fazekas scale based on MRI images. MRI scans were conducted using 3-T scanners (Tim Trio; Siemens AG, Erlangen, Germany). T2-weighted images were utilized to assess the extent of white matter lesion severity following the Fazekas classification system. The Fazekas scale ranges from 0 (no white matter disease) to 3 (severe white matter disease), with only the MRI slice displaying the most pronounced white matter lesions being rated.^27^

###  Plasma BDNF Measurement

 Blood samples from all subjects were collected; plasma was separated by centrifugation at 2000 g for 10 minutes at 4 °C, aliquoted and stored at −80 °C until use in 0.2-mL tubes’ strips. Plasma BDNF level was assessed using the Human BDNF Elisa kit (SIGMA, USA) according to the manufacturer’s recommendations. All samples were analyzed in duplicate, and average values were used for statistical analyses. Appropriate positive and negative controls were included on each plate to ensure assay reliability.

###  Data Analysis

 All categorical variables were presented as number and percentage and continuous variables were presented as mean and standard deviation (SD). Baseline characteristics were compared between patients with and without stroke by chi-square or t-student test for categorical and continuous variables, respectively. Distribution of dependent variables were evaluated using normal p-p plot and the plots demonstrated that the distribution of MoCA and BDNF are not far from normal. Therefore, we used the simple and multiple linear regression model to evaluate the crude and adjusted effect of stroke and its characteristics on MoCA and BDNF. Since WMHs grade was recorded as an ordinal variable with three levels, the ordered probit regression model was used for this variable. All analyses were performed in STATA 14 with *P* values less than 0.05 considered statistically significant.

## Results

 Overall, 228 patients were recruited in the analysis, with a mean age of 63.8 years (minimum 52 and maximum 83 years old). Stroke was found in 52.6% (n = 120). Stroke patients were more commonly female, but there was no difference between patients with and without stroke in age or education (Table 1). Among stroke patients, the lesion was larger than 10mm in 86 (71.7%) patients and involved the temporal lobe in 36 (30.0%) patients.

**Table 1:** Baseline Characteristics Stratified by Stroke Status

**Characteristics**	**Stroke Patients** **(N=120)**	**Control Group** **(N=108)**	***P *** **value**
Age, Mean ± SD	63.6 ± 7.2	64.0 ± 8.2	0.694
Sex, N (%)			
Male	42 (35.0)	54 (50.0)	0.022
Female	78 (65.0)	54 (50.0)	
Education			
Illiterate	82 (68.3)	62 (57.4)	0.088
Literate	38 (31.7)	46 (42.6)	
Residence			
Rural	92 (76.7)	88 (81.5)	0.373
Urban	28 (23.3)	20 (18.5)	
History of hypertension			
Yes	78 (67.2)	48 (44.4)	0.001
No	38 (32.8)	60 (55.6)	
History of diabetes			
Yes	8 (6.9)	5 (4.6)	0.468
No	108 (93.1)	103 (95.4)	

**Figure 1:**
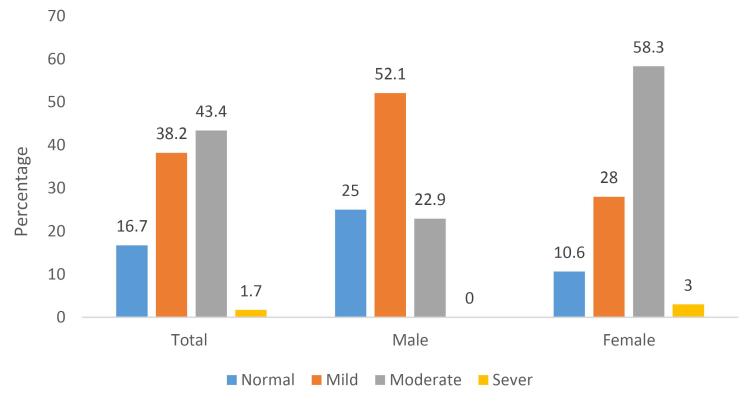
Percentage of Participants in Each MoCA Score Category by Overall Sample and Sex

 In total, 38 patients (16.7%) had a normal MoCA score. Mild and moderate cognitive dysfunction were observed in 87 (38.2%) and 99 (43.4%) patients, respectively. Among males, the majority were classified as having mild cognitive impairment (50 patients, 52.1%), whereas most females fell into the moderate impairment category (77 patients, 58.3%) (Figure 1).

 Stroke patients had lower MoCA score than others, and the effect remained significant after adjustment for age, sex, education and type of residency (adjusted regression coefficient (RC) (95% CI) = -4.0 (-5.0 to -3.0)). In stroke patients, individuals with a stroke size greater than 10 mm had a lower cognitive function score [adjusted RC (95% CI) = -2.2 (-3.8 to -0.6)]; also, patients who had experienced stroke in the temporal lobe had lower MoCA than patients who had experienced stroke in the frontal or parietal lobe [adjusted RC (95% CI) = -2.7 (-4.5 to -0.9)] (Table 2). Further adjustment for history of hypertension and diabetes had a slight influence on the effects.

**Table 2:** Effect of Stroke and its Characteristics on MoCA

**Stroke and its characteristics**		**Number**	**MoCA, Mean±SD**	**Crude RC (95%CI)**	***P*** ** value**	**Adjusted RC**^1^** (95%CI) **	***P*** ** value**	**Adjusted RC**^2^** (95%CI)**	***P*** ** value**
Stroke	No	108	19.2 ± 5.3	Ref.		Ref.		Ref.	
	Yes	120	14.7 ± 5.3	-4.5 (-5.9 to -3.1)	< 0.001	-4.0 (-5.0 to -3.0)	< 0.001	-3.7 (-4.7 to -2.6)	< 0.001
Size	≤ 10 mm	34	15.5 ± 6.2	Ref.		Ref.		Ref.	
	> 10 mm	86	14.3 ± 4.9	-1.1 (-3.2 to 1.0)	0.299	-2.2 (-3.8 to -0.6)	0.009	-2.3 (-3.9 to -0.7)	0.005
Location	Frontal or parietal	84	16.3 ± 5.8	Ref.		Ref.		Ref.	
	Temporal	36	12.6 ± 3.9	-3.7 (-5.8 to -1.6)	0.001	-2.7 (-4.5 to -0.9)	0.004	-3.1 (-5.1 to -1.2)	0.002

RC, Regression coefficient. Adjusted RC^1^: Adjusted for age, sex, education, type of residence. Adjusted RC^2^: Further adjusted for hypertension and diabetes.

 The mean ± SD of BDNF level was 15.9 ± 3.8 in patients without stroke, whereas, it was 12.7 ± 3.9 in patients who experienced a stroke [adjusted RC (95% CI) = -3.2 (-4.2 to -2.2)]. In stroke patients, BDNF was in a lower level when stroke had occurred in the temporal lobe in comparison with the frontal or parietal lobe [adjusted RC (95% CI) = -1.7 (-3.1 to -0.3)], but the size of stroke had no effect on the level of BDNF, and further adjustment for history of hypertension and diabetes had no effects on the results (Table 3).

**Table 3:** Effect of Stroke and its Characteristics on BDNF

**Stroke and its characteristics**		**Number**	**MoCA, Mean±SD**	**Crude RC (95%CI)**	***P*** ** value**	**Adjusted RC**^1^** (95%CI) **	***P*** ** value**	**Adjusted RC**^2^** (95%CI)**	***P*** ** value**
Stroke	No	108	15.9 ± 3.8	Ref.		Ref.		Ref.	
	Yes	120	12.7 ± 3.9	-3.2 (-4.2 to -2.2)	< 0.001	-3.2 (-4.2 to -2.2)	< 0.001	-3.2 (-4.3 to -2.2)	< 0.001
Size	≤ 10 mm	34	12.8 ± 4.6	Ref.		Ref.		Ref.	
	> 10 mm	86	12.7 ± 3.7	-0.1 (-1.7 to 1.5)	0.915	-0.5 (-2.1 to -1.1)	0.563	-0.4 (-2.1 to 1.3)	0.631
Location	Frontal or parietal	84	13.1 ± 3.9	Ref.		Ref.		Ref.	
	Temporal	36	10.9 ± 2.7	-2.1 (-3.5 to -0.7)	0.003	-1.7 (-3.1 to -0.3)	0.017	-1.8 (-3.4 to -0.2)	0.009

RC, Regression coefficient. Adjusted RC^1^: Adjusted for age, sex, education, type of residence. Adjusted RC^2^: Further adjusted for hypertension and diabetes.

 There was not any patient without white matter disease in our study. From 108 patients without stroke, 84 (78%), 16 (15%), and 8 (7%) patients were in the mild, moderate, and severe grade of WMHs, respectively. On the other hand, among 120 patients with stroke, 48 (40%), 40 (33%), and 32 (27%) patients were in the mild, moderate, and severe grade of WMHs, respectively.

 Patients with stroke had significantly higher grades of WMHs [adjusted RC (95% CI) = 1.2 (0.8 to 1.6)]. Among patients with stroke, the size of stroke did not have any association with the grade of WMHs but patients whose stroke occurred in the temporal lobe had a greater grade of WMHs compared with patients whose stroke occurred in the frontal or parietal lobe (Table 4). Further adjustment for history of hypertension and diabetes did not change the results.

**Table 4:** Effect of Stroke and its Characteristics on WMHs

**Stroke and its characteristics**		**Number**	**MoCA, Mean±SD**	**Crude RC (95%CI)**	***P*** ** value**	**Adjusted RC**^1^** (95%CI) **	***P*** ** value**	**Adjusted RC**^2^** (95%CI)**	***P*** ** value**
Stroke	No	108	1.3 ± 0.6	Ref.		Ref.		Ref.	
	Yes	120	1.9 ± 0.8	1.0 (0.6 to 1.3)	< 0.001	1.2 (0.8 to 1.6)	< 0.001	1.2 (0.8 to 1.6)	< 0.001
Size	≤ 10 mm	34	1.9 ± 0.8	Ref.		Ref.		Ref.	
	> 10 mm	86	1.9 ± 0.8	-0.03 (-0.5 to 0.5)	0.875	0.2 (-0.3 to 0.7)	0.435	0.1 (-0.4 to 0.6)	0.719
Location	Frontal or parietal	84	1.7 ± 0.7	Ref.		Ref.		Ref.	
	Temporal	36	2.3 ± 0.7	0.9 (0.4 to 1.3)	< 0.001	0.7 (0.2 to 1.2)	0.004	0.5 (0.1 to 1.1)	0.037

RC, Regression coefficient. Adjusted RC^1^: Adjusted for age, sex, education, type of residence. Adjusted RC^2^: Further adjusted for hypertension and diabetes.

## Discussion

 Stroke is a cerebrovascular disease and a common cause of brain lesions that can result in severe and long-lasting damage to the brain, leading to cognitive impairment, WMHs, and altered levels of circulating biomarkers such as BDNF.^28^ In light of these observations, our study set out with the intention to comprehensively compare cognitive function, WMH scores, and plasma BDNF levels between patients with clinical symptoms of stroke with or without a history of stroke.

 In the present study, we investigated a group of 228 patients with clinical symptoms of stroke. Through the use of MRI, we identified 120 patients with a brain lesion compatible with stroke and 108 without such a brain lesion. Comparative analyses were conducted to assess cognitive function (measured by MoCA scores), WMH (quantified using Fazekas scores), and plasma BDNF levels between these two groups. Our findings revealed significantly lower MoCA scores and reduced plasma BDNF levels in patients with stroke. Moreover, the burden of WMH was higher in the stroke group. Further analysis within the subgroup with prior strokes showed that lesion size did not affect cognitive function, in contrast to WMH or circulating BDNF levels. However, lesion location demonstrated significant associations with all measures. Frontal or parietal lesions were associated with higher MoCA scores and elevated BDNF levels but lower Fazekas scores pertained to temporal lesions. These results enhance our understanding of neurocognitive changes following strokes and provide insight for future research opportunities in this field.

 Our findings demonstrate a significant elevation in WMH scores among patients with a history of stroke, affirming previous investigations that report an augmented prevalence and severity of white matter lesions subsequent to ischemic events such as strokes.^29^ Numerous studies have employed Fazekas scores to explore the association between WMH and cognitive impairment. For instance, Verdelho et al conducted an investigation revealing a positive correlation between elevated Fazekas scores and an increased risk of cognitive decline and dementia among elderly individuals.^30^ Furthermore, Sachdev et al revealed that Fazekas scores were considerably associated with inferior performance on cognitive tests, even after controlling for age, gender, and other confounding variables.^31^ These findings suggest that Fazekas scores can be a valuable tool for predicting cognitive impairment. The precise mechanisms underlying alterations in Fazekas scores following stroke remain incompletely understood; however, it is possible to consider their association with disruptions to the blood-brain barrier (BBB).^32^ In addition to the alterations in Fazekas scores due to stroke, changes in Fazekas scores over time may have prognostic implications for individuals affected by strokes. Notably, a progressive increase in Fazekas scores over time has been linked to an augmented risk of recurrent stroke and increased mortality among stroke patients.^32^

 The MoCA has been widely used as a screening tool for identifying cognitive dysfunction among stroke patients. Our findings revealed a significant reduction in MoCA scores among individuals with stroke, corroborating prior investigations that reported an escalated prevalence and severity of cognitive decline following stroke.^33,34^ Overall, the MoCA is a valuable screening tool for identifying cognitive impairment, and notably, since its inception, the MoCA has undergone several revisions aimed at enhancing its validity and reliability.^35^ A study conducted by Salvadori et al observed that stroke patients exhibited significantly lower MoCA scores compared to healthy individuals, and these scores were found to be inversely correlated with the severity of strokes. Based on their findings, the authors propose that MoCA holds promise as a valuable tool for evaluating cognitive impairment in stroke patients while also serving as an effective means of monitoring cognitive changes over time.^36^ The timing of MoCA assessment following a stroke event appears to influence the obtained scores. A separate investigation conducted by Patel et al revealed that MoCA scores demonstrated improvement in stroke patients over time. The authors suggest that cognitive rehabilitation interventions may improve cognitive function among stroke patients.^37^ Various factors may contribute to changes in MoCA scores following a stroke event. A study by Yang et al found that MoCA scores were significantly lower in stroke patients with depression.^38^ Another investigation carried out by Demeyere et al demonstrated that stroke patients with left hemisphere lesions exhibited lower MoCA scores than those with right hemisphere lesions. This disparity suggests that the left hemisphere may play a more important role in cognitive function.^39^ It is worth noting that additional research is warranted to determine the optimal timing of MoCA assessment after stroke and to evaluate the effectiveness of cognitive rehabilitation interventions in improving cognitive outcomes.

 BDNF, a vital protein involved in neuronal growth, survival, and synaptic plasticity, has garnered considerable attention due to its potential role in post-stroke outcomes. Emerging evidence suggests that BDNF levels may be altered following stroke, potentially contributing to cognitive dysfunction and other neurological deficits.^40^ Our study yielded significant insight as we observed lower plasma BDNF levels among patients with stroke. This finding aligns with previous investigations reporting diminished circulating BDNF levels following ischemic injury such as strokes.^41,42^ These findings collectively underscore the significance of investigating the impact of altered BDNF levels on post-stroke pathophysiology and highlight its potential as a biomarker for assessing disease progression and therapeutic interventions.

 The occurrence of stroke exerts profound effects on brain neurochemistry, leading to notable alterations in various molecular pathways. In a recent investigation, Øverberg et al reported significantly lower plasma BDNF levels in acute stroke patients compared to healthy controls.^43^ Similarly, another study by Rodier et al reported a negative correlation between plasma BDNF levels and the severity of strokes.^10^ These findings underscore the potential utility of circulating BDNF as an accessible biomarker for assessing stroke pathophysiology and prognosis. In addition to stroke, changes in BDNF expression have been observed in other neurological disorders such as Alzheimer’s disease, Parkinson’s disease, and depression.^44^ This highlights the multifaceted role played by BDNF across different pathological conditions affecting the central nervous system. Further investigations are warranted to elucidate the precise mechanisms underlying these observed changes in BDNF expression and their implications for diagnosis, treatment strategies, and therapeutic interventions targeting these neurological disorders. The findings from various studies strongly support the notion that alterations in BDNF expression following stroke play a significant role in the subsequent recovery process. Hence, BDNF represents a promising therapeutic target for stroke treatment and rehabilitation.^9^ BDNF may be a common molecular target for the development of new therapeutic interventions for neurological disorders.^45^ Therefore, understanding the mechanisms underlying BDNF regulation in these diseases may provide new insight into the pathogenesis of these disorders and help develop new therapeutic strategies.

 Our comprehensive analysis did not yield any significant correlation between lesion size and patient outcomes across various measures, encompassing MoCA scores, Fazekas scores, and circulating BDNF levels. However, an effect was observed based on the specific location of the lesion within the brain. Notably, patients with frontal or parietal lesions exhibited significantly higher MoCA scores and elevated circulating BDNF levels in comparison to those with temporal lesions. Conversely, patients with temporal lesions demonstrated lower Fazekas scores indicative of increased severity in white matter lesion burden. These findings underscore the importance of considering lesion location as a potential determinant for cognitive performance outcomes and alterations in BDNF expression following stroke events.

 Our study findings provide compelling evidence supporting the utility of employing a multi-biomarker approach, encompassing measures such as the MoCA score, Fazekas score, and circulating BDNF levels, to gain valuable insight into the neurological status of post-stroke patients. Overall, these results contribute to the growing knowledge on neurocognitive changes following cerebrovascular events such as strokes and highlight potential avenues for improving patient outcomes through early detection and intervention strategies that should be further studied.

 One notable limitation of this study is its cross-sectional design, which prevents the establishment of causal relationships between stroke history and neurobiological outcomes. To better understand the temporal progression of these changes, it is recommended that future investigations involve follow-up assessments of participants in the Golestan Cohort Study, with repeated measurements of MoCA, BDNF, and WMH at extended intervals. Moreover, Participants were selected exclusively from the Golestan province in north-eastern Iran, and most of them also lived in rural areas. Cultural, genetic, environmental, and healthcare-access factors unique to this region may influence cognitive outcomes, WMH burden, and BDNF levels, limiting generalizability. In addition, every subject exhibited at least mild WMH, so the findings may not extend to stroke populations without WMH or to neurologically healthy controls.

## Conclusion

 In conclusion, our research establishes robust connections between ischemic brain injury such as stroke and altered cognitive function, WMH burden, and perturbed plasma BDNF levels. These findings highlight both the importance of continued research in this area and suggest that incorporating biomarker assessments such as BDNF measurements or non-invasive imaging techniques assessing WMH could offer valuable information regarding post-stroke patients’ neurological status. Ultimately, these results make significant contributions towards advancing our understanding of neurocognitive changes following cerebrovascular events while simultaneously paving the way for innovative strategies aimed at improving patient outcomes through early detection and targeted interventions.
